# The efficacy of screening with FDG-PET/CT for distant metastases in breast cancer patients scheduled for neoadjuvant systemic therapy

**DOI:** 10.1007/s10549-024-07478-5

**Published:** 2024-09-26

**Authors:** Jetske L. B. Gunster, Frederieke H. van Duijnhoven, Astrid N. Scholten, Carolien H. Smorenburg, Vincent O. Dezentje, Josefien P. van Olmen, Corrie A. M. Marijnen, Marcel P. M. Stokkel, Claudette E. Loo, A. Marjolein Schrijver

**Affiliations:** 1 Department of Radiation Oncology, Netherlands Cancer Institute-Antoni van Leeuwenhoek, Amsterdam, Plesmanlaan 121, 1066 CX The Netherlands; 2 Department of Radiation Oncology, Leiden University Medical Center, Leiden, The Netherlands; 3 Department of Surgical Oncology, Netherlands Cancer Institute-Antoni Van Leeuwenhoek, Amsterdam, The Netherlands; 4 Department of Medical Oncology, Netherlands Cancer Institute-Antoni Van Leeuwenhoek, Amsterdam, The Netherlands; 5 Department of Nuclear Medicine, Netherlands Cancer Institute-Antoni Van Leeuwenhoek, Amsterdam, The Netherlands; 6 Department of Radiology, Netherlands Cancer Institute-Antoni Van Leeuwenhoek, Amsterdam, The Netherlands

**Keywords:** Breast cancer, Neoadjuvant systemic therapy, FDG-PET/CT, Distant metastases

## Abstract

**Purpose:**

This study aims to identify which breast cancer patients benefit from the routine use of FDG-PET/CT in a large cohort of patients scheduled for neoadjuvant systemic therapy (NST).

**Methods:**

A total of 1337 breast cancer patients eligible for NST were identified from a retrospective database between 2011 and 2020 at a single tertiary care hospital. All patients underwent staging with FDG-PET/CT prior to NST. The incidence and extent of asymptomatic distant metastases in different patient subgroups were determined, as well as the impact on treatment. Logistic regression analysis was used to identify prognostic patient and tumor characteristics.

**Results:**

FDG-PET/CT detected distant metastases in 109 patients (8%). Initial clinical stage was a prognostic factor for the presence of distant metastases, with a significantly higher risk for stage 2b and 3 as opposed to lower stages (p < 0.001). The incidence of distant metastases was 3% (4/125) for stage 1, 2% (8/534) for stage 2a, 7% (24/354) for stage 2b and 23% (73/324) for stage 3. Other characteristics such as age, tumor subtype, histological type and grade were not correlated with the risk of distant metastases. Among the subset of patients with distant metastases, 46% received palliative treatment, while the remaining 54% were diagnosed with oligometastatic breast cancer and were treated with curative intent.

**Conclusion:**

The results of the current study support the routine use of FDG-PET/CT for the detection of distant metastases in breast cancer patients with initial clinical stage 2b and 3, regardless of tumor subtype.

## Introduction

Breast cancer is the most commonly diagnosed malignancy among women worldwide [1]. Accurate evaluation of the extent of disease is essential before initiating treatment, as it impacts prognosis and treatment strategy. Early detection of distant metastases may change curative into palliative therapy, reducing treatment burden for patients.

In early stage breast cancer, initial staging is primarily focused on evaluating the extent of locoregional disease [2, 3]. While international clinical guidelines acknowledge the need for additional screening for distant metastases in patients with certain clinical features, there is considerable variation in recommendations between guidelines. For example, the ESMO Clinical Practice Guidelines recommend to consider screening for distant metastases in patients with suggestive symptoms, clinically positive axillary nodes, large tumors (≥ 5 cm) or aggressive biology [2]. On the other hand, the NCCN Clinical Practice Guidelines in Oncology advise screening for distant metastases in patients with either suggestive signs or symptoms or in case of unresectable, clinical stage 3 disease [3]. This lack of consensus makes it challenging to establish a uniform approach for identifying distant metastases in breast cancer patients.

In addition, there is ongoing debate regarding the choice of imaging modality. For the detection of distant metastases, 18F-fluorodeoxyglucose positron emission tomography coupled with computed tomography (FDG-PET/CT) has shown high accuracy compared to other imaging techniques [4–7]. However, conducting routine FDG-PET/CT in early stage breast cancer patients may result in high rates of incidental findings and increased healthcare costs[8, 9]. Consequently, the decision to utilize FDG-PET/CT in the initial staging of breast cancer patients requires careful consideration, as the potential benefits need to outweigh the downsides.

Clarification on the tailored use of FDG-PET/CT is necessary, particularly for breast cancer patients scheduled for neoadjuvant systemic therapy (NST). The identification of metastatic disease can prompt a shift in treatment strategy towards palliative care, thereby avoiding unnecessary treatment. Additionally, advancements in the treatment of oligometastatic breast cancer emphasize the critical role of accurate staging at time of diagnosis.

This study aims to identify which breast cancer patients benefit from the routine use of FDG-PET/CT, by analyzing a large cohort of patients scheduled for NST at our institute.

## Materials and methods

### Study design and participants

All patients with breast cancer scheduled for NST were identified from a retrospective database at the Netherlands Cancer Institute. The indication for NST was determined according to (inter)national guidelines incorporating information on age, clinical stage, tumor size and histological type. Data of patients scheduled for NST between January 2011 and December 2020 were collected.

An FDG-PET/CT scan preceded by routine conventional imaging of the primary tumor and locoregional lymph nodes was required for inclusion. Conventional imaging for locoregional staging consisted of a mammography, ultrasound of the breast and regional lymph nodes and breast MRI. Exclusion criteria were recurrent breast cancer, axillary surgery performed prior to FDG-PET/CT and suspected metastatic disease based on clinical symptoms. In case of bilateral breast cancer, the tumor with the worst prognosis was included in the analysis.

This study was approved by the Institutional Review Board of the Netherlands Cancer Institute-Antoni van Leeuwenhoek.

### Imaging

Before the FDG-PET/CT scan, blood glucose levels of patients were required to be lower than 10 mmol/L. A weight-adjusted dose of 180–240 MBq 18F-FDG was administered intravenously. Approximately 1 h after administration, FDG-PET/CT scanning (Philips Gemini TF Big Bore, Cleveland, OH, USA) was performed with acquisitions from the base of the skull to the groin region for regional staging and detection of distant metastases. This involved a low-dose CT scan without contrast to correct for attenuation. Additional PET/CT images in prone position were acquired for the assessment of uptake in the primary tumor.

Increased FDG uptake, exclusively seen on FDG-PET/CT and not corresponding to physiological uptake patterns or pre-existent activity, was classified as an additional lesion. Densities seen on low-dose CT scan were classified as additional lesions as well. All scans were interpreted by experienced nuclear medicine physicians with access to relevant clinical patient data. The standard clinical reads were used for analysis and each scan was read by a single nuclear medicine physician.

### Distant metastases

Additional distant lesions were further evaluated using targeted imaging procedures and, preferably, histopathological verification. Distant metastases detected within six months of follow-up after negative FDG-PET/CT were considered as occult metastases.

### Outcomes

The primary objective was to determine the incidence of asymptomatic distant metastases using FDG-PET/CT. Overall incidence was determined, as well as stage and tumor subtype specific incidence. Clinical stage was classified according to the eighth edition of the AJCC system [10] and a distinction was made between patients with stage 2a and 2b. Tumor subtype was classified as luminal (ER + and/or PR + , HER2-), HER2-positive (ER ± and/or PR ± , HER2 +) or triple-negative (ER-, PR-, HER2-). Extent of metastatic disease was classified as 1–3, 3–5 or more than 5 lesions. Treatment modifications were recorded, including transitions to palliative care, or in case of limited disease, to curative-intent therapy targeting metastatic lesions.

The secondary objective was to assess the association between clinical and pathological characteristics and the detection of distant metastases with FDG-PET/CT. High-risk characteristics such as young age, advanced clinical stage, HER2-positive or triple-negative subtype and high-grade tumors were considered relevant for analysis.

Prior to analysis, we considered an incidence of asymptomatic distant metastases above 3% to be sufficiently high to justify the routine use of FDG-PET/CT.

### Statistical analysis

Descriptive and explorative analyses were performed with the use of statistical software R version 4.3.0. Data were expressed as means for continuous variables and proportions for categorical variables. The threshold for statistical significance was set at p < 0.05. Frequencies and proportions of distant metastases detected with FDG-PET/CT were calculated and reported as percentages with 95% confidence intervals. Associations between patient and tumor characteristics and distant metastases were assessed with logistic regression analysis. In case of missing values for categorical variables, the missing values were tested for randomness and excluded from analysis if appropriate. Reference levels in the logistic regression analysis required a minimum number of five events.

## Results

### Patient characteristics

**Fig. 1:**
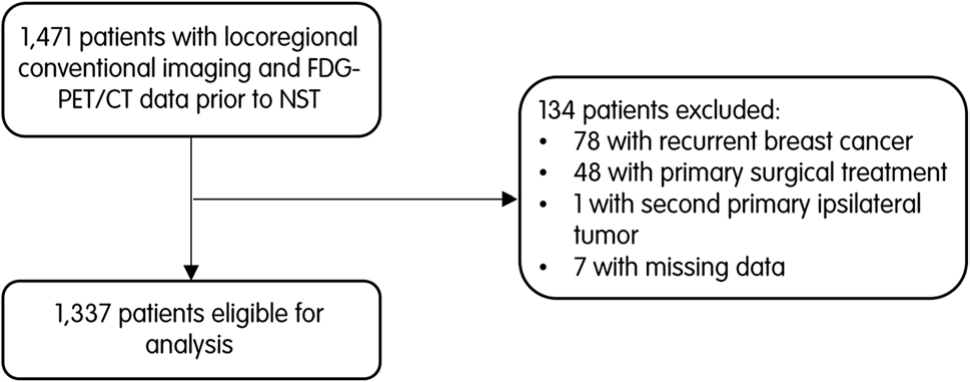
Flowchart depicting the exclusion of patients from the current analysis

A total of 1471 patients scheduled for NST between 2011 and 2020 had FDG-PET/CT data available. Of these patients, 1337 met the inclusion criteria and were eligible for analysis (Fig. 1). The mean age was 50 years. The majority of patients had clinical stage 2a (40%) disease. Tumors of the luminal subtype were most common (47%). Most patients had grade 2 (49%) or 3 (44%) disease. An overview of the baseline characteristics is shown in Table 1.

**Table 1:** Clinical and pathological baseline characteristics of all patients. Abbreviations: *NST* non-specific type, *ILC* invasive lobular carcinoma

	All patients (n = 1337)	
**Age, mean (SD), years**	50	12
**Female gender**	1335	100%
**Screen detected**	251	19%
**Unilateral breast cancer**	1284	96%
**Unifocal tumor**	912	68%
**Clinical stage prior to FDG-PET/CT**		
1	125	9%
2a	534	40%
2b	354	27%
3	324	24%
**Histology**		
NST	1145	86%
ILC	154	11%
Other	36	3%
Missing	2	0%
**Subtype**		
Luminal	634	47%
HER2 +	342	26%
Triple-negative	361	27%
**Grade**		
1	55	4%
2	659	49%
3	583	44%
Missing	40	3%
**Ki-67**		
≤ 10%	297	22%
> 10%	990	74%
Missing	50	4%
**FDG-avid tumor**	1287	96%

### Distant metastases

A total of 109 patients were diagnosed with asymptomatic distant metastases following FDG-PET/CT. Thus, the overall incidence of distant metastases was 8% [95%CI 7–10%].

Histopathological confirmation of metastatic disease was obtained in 51 patients (47%). In the remaining 58 patients (53%), final diagnosis was based on imaging, primarily FDG-PET/CT, and often supplemented with an additional CT and/or MRI scan. This could be due to inconclusive histopathological samples, the inaccessibility of lesions for biopsy, or because imaging alone was highly suggestive for metastatic disease. All cases were thoroughly reviewed and discussed in multidisciplinary meetings, where imaging findings and clinical context were carefully considered before establishing a final diagnosis.

In the 109 patients with distant metastases, the most common sites were bone (63%), distant lymph nodes (34%), liver (23%) and lung (21%). Less common sites included the endocrine system (2%), the gastrointestinal (1%) and urogenital (1%) tract or other remaining sites (1%).

One patient developed distant metastases within 6 months after a negative FDG-PET/CT.

### Stage and subtype specific incidence of distant metastases

The rate of distant metastases was 3% [95%CI 1–8%] for patients with clinical stage 1 and 2% [1–3%] for stage 2a. In patients with stage 2b and 3, these rates increased to 7% [4–10%] and 23% [18–28%], respectively.

Between tumor subtypes, rates for distant metastases were similar; 9% [95%CI 7–12%] for luminal, 9% [6–12%] for HER2-positive, and 6% [3–8%] for triple-negative tumors.

**Fig. 2:**
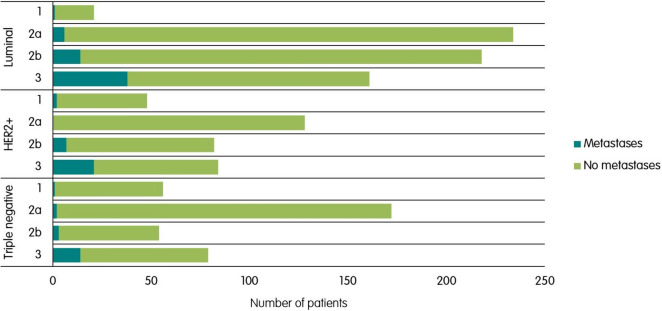
Distribution of distant metastases in patients, stratified by tumor subtype and clinical stage

In luminal subtype patients, rates were 5% [95%CI 0–2%] for stage 1, 3% [1–6%] for stage 2a, 7% [4–11%] for stage 2b and 31% [17–31%] for stage 3. For patients with HER2-positive tumors, these numbers were 4% [1–14%], 0% [0–3%], 9% [4–17%] and 25% [16–36%], and for patients with triple-negative tumors, these rates were 2% [0–10%], 1% [0–4%], 6% [1–15%] and 18% [10–28%], respectively. Figure 2 displays the number of patients with distant metastases per subtype, categorized by stage.

### Treatment of distant metastases

Among the 109 patients with distant metastases, therapy shifted from curative to palliative intent in 50 patients (46%). In 46 of these patients, metastatic disease was widespread (i.e., more than 5 lesions were detected). The remaining 4 patients initially presented with distant metastases confined to 3–5 lesions, but clinicians made the decision not to pursue curative treatment. This decision was influenced by factors such as advanced age, significant comorbidity and unresponsiveness to systemic therapy.

In 56 patients (51%), there was a limited number of metastatic lesions, classified as oligometastatic breast cancer. Specifically, 29 patients (52%) had a single lesion, 22 patients (39%) had 2 and 2 patients (4%) had 3 lesions. The remaining 3 patients (5%) had 3–5 metastatic lesions. All these patients underwent curative-intent treatment tailored to their condition.

There were 3 patients (3%) with distant metastases who chose not to proceed with treatment. These patients either sought alternative therapy or declined treatment.

Table 2 provides a summary of patients with distant metastases who underwent treatment with either palliative or curative intent.

**Table 2:** Summary of patients with distant metastases by clinical stage and tumor subtype, categorized by curative and palliative treatment intent.  Abbreviations: *DM* distant metastases

	Total of DM		Curative treatment		Palliative treatment	
	n	%	n	%	n	%
**Clinical stage**						
1	4	3%	4	100%	0	0%
2a	8	2%	7	88%	1	13%
2b	24	7%	11	46%	13	54%
3	73	23%	34	47%	38	53%
**Subtype**						
Luminal	59	9%	28	48%	30	52%
HER2 +	30	9%	16	52%	15	48%
Triple-negative	20	6%	12	60%	8	40%

### Patient and tumor characteristics associated with distant metastases

In univariable analysis (Table 3), clinical stage 1, 2a and 2b were associated with a significantly lower risk of detecting distant metastases as compared to patients with clinical stage 3 (p < 0.001). Comparison between subgroups 2a and 2b showed a significantly higher risk of distant metastases for patients with stage 2b (OR 4.78, [95%CI 2.12–10.77], p < 0.001). Furthermore, triple-negative subtype showed a significantly lower risk of distant metastases as compared to luminal subtype (p = 0.037). No significant associations between the risk of distant metastases and age, histological type and grade were found in univariable analysis.

**Table 3:** Univariable and multivariable logistic regression analysis. Statistically significant values are displayed in bold formatting (p < 0.05). Abbreviations: *NST* non-specific type, *ILC* invasive lobular carcinoma

	Univariable analysis			Multivariable analysis		
	OR	95% CI	P value	OR	95% CI	P value
**Age**	1.01	0.99–1.03	0.258	-	-	-
**Clinical stage**						
1	0.11	0.04–0.32	**< 0.001**	0.12	0.04–0.34	**< 0.001**
2a	0.05	0.03–0.11	**< 0.001**	0.05	0.03–0.11	**< 0.001**
2b	0.25	0.15–0.41	** < 0.001**	0.24	0.15–0.40	** < 0.001**
3	ref	-	-	ref	-	-
**Subtype**						
Luminal	ref	-	-	ref	-	-
HER2 +	0.94	0.59–1.49	0.782	1.00	0.61–1.63	0.999
Triple-negative	0.57	0.34–0.97	**0.037**	0.68	0.39–1.18	0.168
**Histology**						
NST	ref		-	-	-	-
ILC	1.38	0.79–2.41	0.265	-	-	-
Other	1.48	0.51–4.29	0.467	-	-	-
**Grade**						
1	0.49	0.11–2.01	0.328	-	-	-
2	1.34	0.89–2.01	0.163	-	-	-
3	ref	-	-	-	-	-

In multivariable analysis, the association between advanced stages of disease and distant metastases remained significant (p < 0.001). No other significant associations were demonstrated in multivariable analysis (Table 3).

## Discussion

This study investigated the value of screening for distant metastases with FDG-PET/CT in a large cohort of 1337 breast cancer patients. Our findings support the use of FDG-PET/CT for identifying asymptomatic distant metastases in patients with clinical stage 2b and 3, as the incidence in these groups exceeded our preset threshold value of 3%. Furthermore, as clinical stage advanced, there was a notable rise in patients shifting to palliative therapy, highlighting the significance of FDG-PET/CT in these groups.

The value of FDG-PET/CT for detecting asymptomatic distant metastases has been addressed in previous smaller studies. Dayes et al. conducted a randomized trial involving 369 patients with stage 2b and 3 breast cancer, comparing FDG-PET/CT with conventional imaging [11]. They found that FDG-PET/CT detected distant metastases in 23% of patients, whereas conventional imaging identified distant metastases in 11%. The authors therefore recommend the use of FDG-PET/CT in clinical stage 2b and 3 breast cancer, which aligns with our findings. Additionally, a comprehensive review by Groheux et al. reached similar conclusions [12]. Although our study was observational rather than randomized, it includes the largest cohort to date on this topic [13–17]. Moreover, our study included patients with early stage breast cancer as well. Given the current discrepancies in clinical guidelines for these patients, our study significantly contributes to the existing literature on this topic.

We observed that the identification of distant metastases by FDG-PET/CT resulted in a shift in treatment strategy for all patients. Nearly half of the patients with distant metastases received palliative treatment and the other patients underwent more extensive, curative treatment, including local ablative therapy for their metastatic lesions. As a result, FDG-PET/CT led to a more tailored treatment strategy for patients in whom asymptomatic metastatic disease was detected. The treatment options for oligometastatic breast cancer (OMBC) are continuously evolving, with ESMO guidelines advocating a multimodal approach involving systemic therapy in combination with local ablative therapy for metastases [18]. These recommendations are primarily based on phase two trials and cohort studies [19–21]. However, recent randomized trials, such as the NRG-BR002 study, show no significant difference in survival when adding local ablative therapy to standard systemic therapy for OMBC [22, 23]. Therefore, further results from ongoing randomized trials are needed before assessing the role of FDG-PET/CT in the detection of OMBC.

Interestingly, we did not find a significant association between tumor subtype and distant metastases. Triple-negative tumors are known to act more aggressively with an increased likelihood of distant recurrence and death in the first five years after diagnosis compared to other breast cancer subtypes [24–26]. Therefore, it might be expected that these patients more often present with metastatic disease at time of diagnosis. However, this was not confirmed in our study and these findings are supported by others. Ulaner et al. performed a retrospective analysis including 232 patients with stage 1 to 3 triple-negative breast cancer. FDG-PET/CT imaging at time of diagnosis demonstrated distant metastases in 0% of stage 1 patients, 5% of stage 2a, 15% of stage 2b and 33% of stage 3 patients [27]. These percentages are similar to the findings in our cohort, consisting of breast cancer patients with luminal and HER2-positive breast cancer as well. Important to note is that our study excluded patients with clinical evidence of distant metastases prior to FDG-PET/CT. It might be that a relatively higher proportion of patients with triple-negative tumors presented with symptoms suggesting metastatic disease at time of diagnosis. Therefore, we compared our findings with national data from the Netherlands Cancer Registry (NCR) from the same time period [28]. The NCR rates showed that symptomatic, synchronous distant metastases occurred in 6% of patients with triple-negative tumors, compared to 4% with luminal and 8% with HER2-positive tumors. We therefore consider the findings in our study to be representative and conclude that triple-negative tumors are not significantly associated with the risk of distant metastases at time of diagnosis.

To the best of our knowledge, this is the largest study investigating the role of FDG-PET/CT in the workup for breast cancer patients. With an expanding indication for NST, optimization of the diagnostic workup is important and contributes to tailored treatment. Limitations of our study include the fact that it was performed at a single institute. The retrospective design makes it prone for selection and interpretation bias. Data were extracted from FDG-PET/CT reports cited by nuclear physicians. Therefore, findings may have been influenced by subjectivity of the involved physician.

Our study highlights the importance of clinical breast cancer stage when deciding upon the value of FDG-PET/CT. The yield of FDG-PET/CT is higher with the clinical stage increasing, but there is no consensus on a threshold value for the risk of distant metastases to justify the routine use of FDG-PET/CT or other conventional methods for the detection of distant metastases. In daily practice at our institute, physicians generally apply a threshold value of 3%, but this is arbitrary. International guidelines do not include a threshold value and different aspects should be taken into consideration when deciding upon one.

Besides the detection of distant metastases, there are other aspects that should be carefully considered for the implementation of FDG-PET/CT in routine practice. Firstly, FDG-PET/CT may lead to the detection of additional regional lymph node metastases, consequently resulting in upstaging and changes in treatment. Secondly, an important drawback of full-body scans such as FDG-PET/CT include high rates of incidental findings, exposing patients to invasive or non-invasive additional procedures. In addition to patient distress, this may lead to extra morbidity and higher healthcare costs. Given these concerns, future research should focus on evaluating the clinical impact of FDG-PET/CT on locoregional treatment strategies and examining the implications of incidental findings unrelated to breast cancer. Additionally, the overall cost-effectiveness of FDG-PET/CT in this context should be investigated.

In conclusion, the use of FDG-PET/CT for the detection of asymptomatic distant metastases in breast cancer patients shows a substantial value in patients with clinical stage 2b or 3.

## Data Availability

Datasets are available from the corresponding author on reasonable request.
